# Supplementary material for: Insights into the regulation of VPS13 family bridge-like lipid transfer proteins from the structure of VPS13C
Source: bioRxiv. 2025 Nov 11:2025.11.10.687702. Preprint. [Version 1] doi: 10.1101/2025.11.10.687702 (PMC12642616; doi:10.1101/2025.11.10.687702)
Supplement: 1 [file NIHPP2025.11.10.687702v1-supplement-1.pdf]

bioRxiv preprint doi: https://doi.org/10.1101/2025.11.10.687702; this version posted November 11, 2025. The copyright holder for this preprint (which was not certified by peer review) is the author/funder, who has granted bioRxiv a license to display the preprint in perpetuity. It is made available under aCC-BY 4.0 International license.

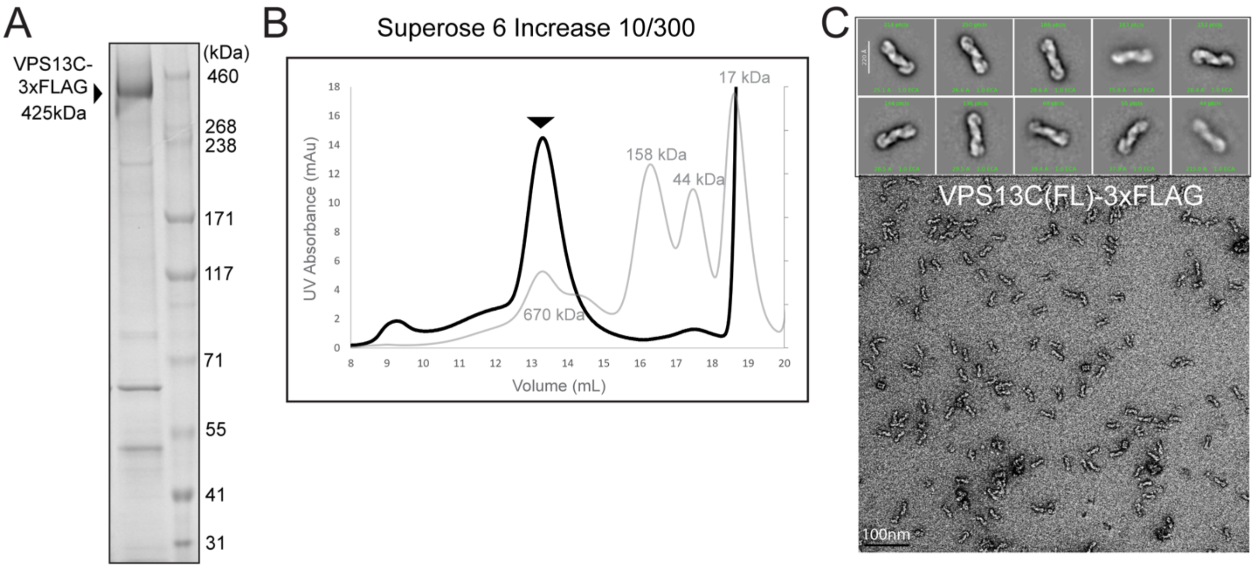

Figure S1. Preparation of full-length VPS13C for Cryo-EM studies.
(A) Purified full-length VPS13C-3xFLAG expressed in Expi293F cells analyzed by SDS-PAGE. (B) Size-exclusion chromatography of VPS13C-3xFLAG (425 kDa) on a Superose 6 10/300 column shows a monodisperse peak eluting near the 670 kDa standard. (C) Representative negative-stain EM image and 2D averages reveal a homogeneous sample with a rod-like shape.

bioRxiv preprint doi: https://doi.org/10.1101/2025.11.10.687702; this version posted November 11, 2025. The copyright holder for this preprint (which was not certified by peer review) is the author/funder, who has granted bioRxiv a license to display the preprint in perpetuity. It is made available under aCC-BY 4.0 International license.

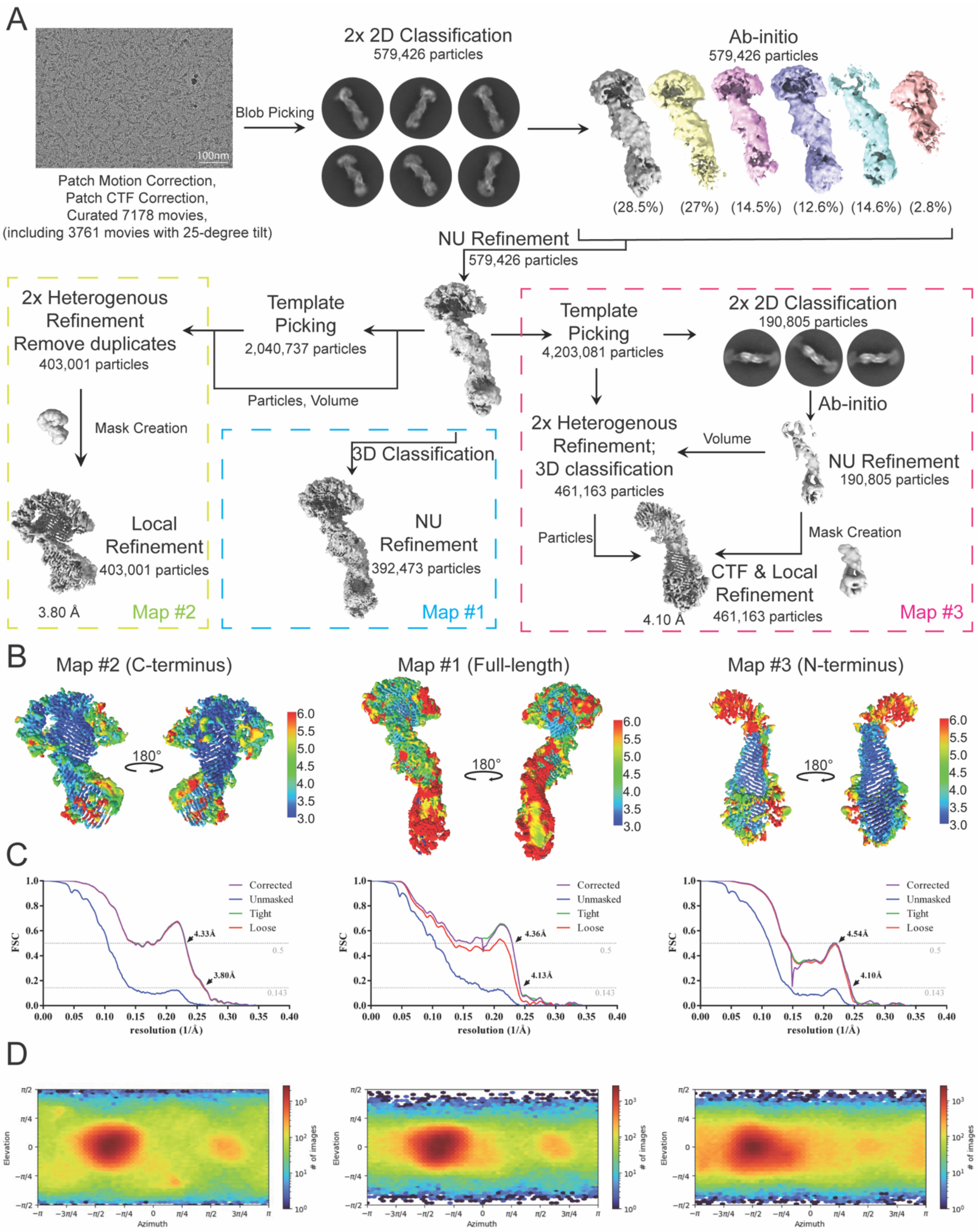

Figure S2. Data processing of full-length VPS13C-3xFLAG.
(A) Workflow of data processing. (B) Local resolution estimation of the three final maps, shown from front and back views. Map #1 is the lower-resolution full-length map; Map #2 and Map #3 are locally-refined maps of the C-terminus and the N-terminus, respectively.(C) FSC curves from final refinements for all three maps. (D) Orientation distribution plots for particles contributing to the three maps, showing preferred orientation.

bioRxiv preprint doi: https://doi.org/10.1101/2025.11.10.687702; this version posted November 11, 2025. The copyright holder for this preprint (which was not certified by peer review) is the author/funder, who has granted bioRxiv a license to display the preprint in perpetuity. It is made available under aCC-BY 4.0 International license.

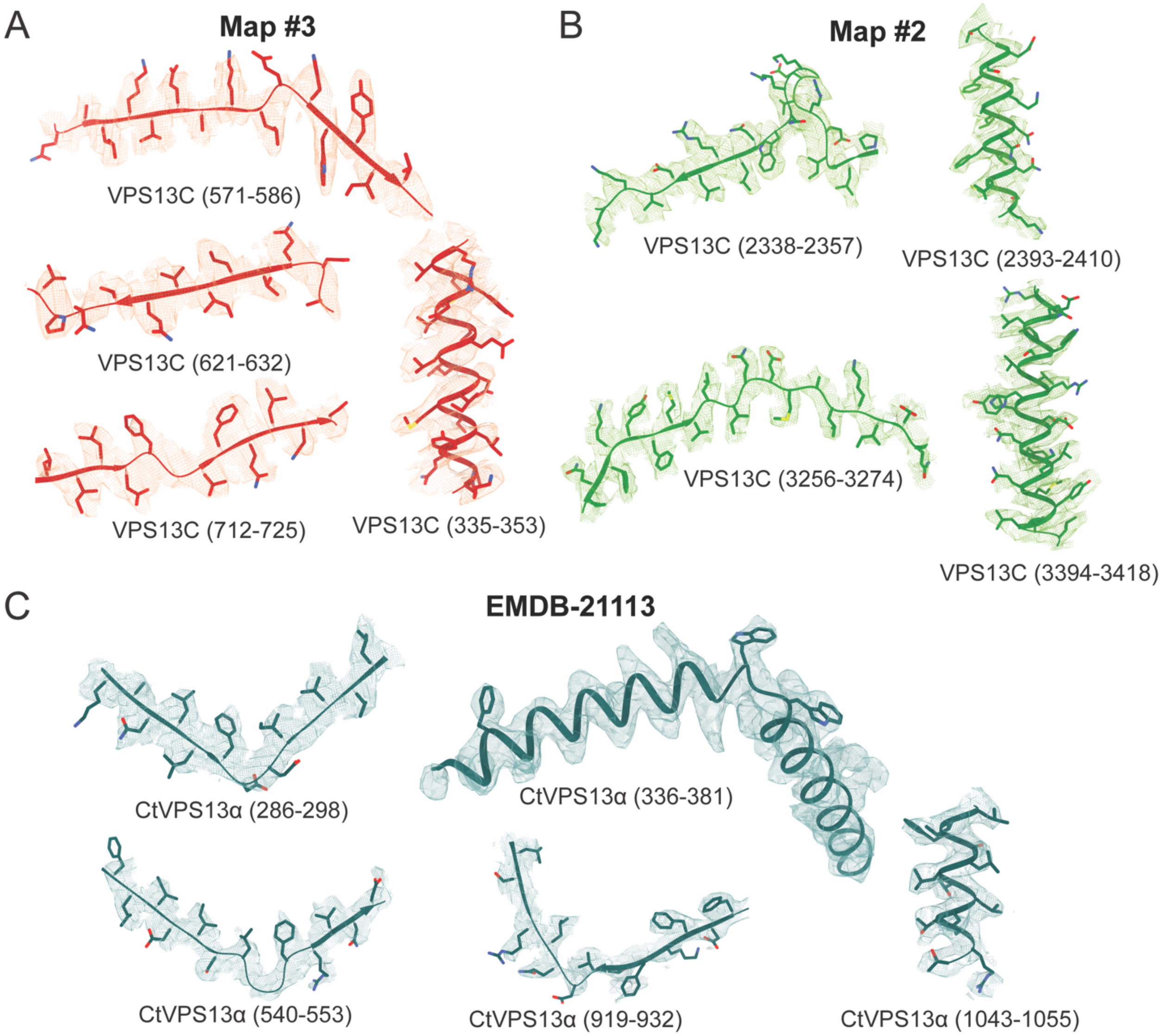

Figure S3. Examples of model fitting into EM maps for VPS13C and *Ct*VPS13α.
(A) Representative map and model fits for regions in the VPS13C's N-terminus. (B) Representative map and model fits for regions in the VPS13C's C-terminus.(C) Representative map and model fits for regions in *Ct*VPS13α.

bioRxiv preprint doi: https://doi.org/10.1101/2025.11.10.687702; this version posted November 11, 2025. The copyright holder for this preprint (which was not certified by peer review) is the author/funder, who has granted bioRxiv a license to display the preprint in perpetuity. It is made available under aCC-BY 4.0 International license.

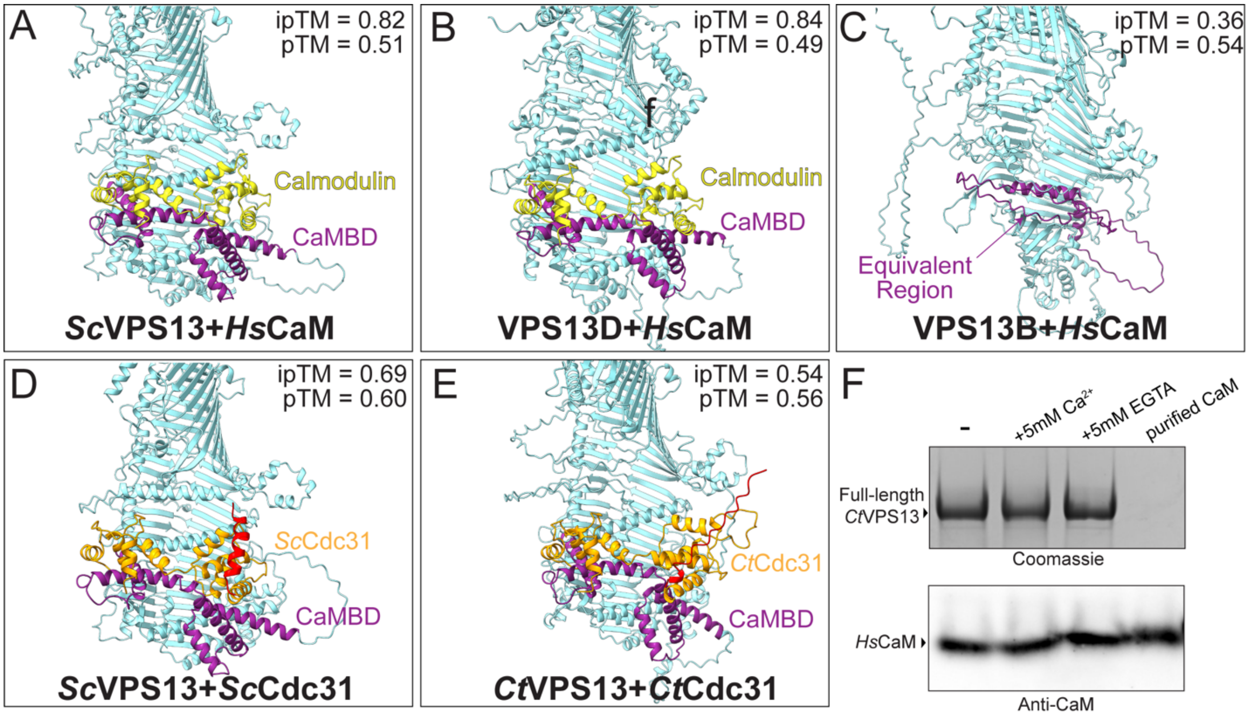

Figure S4. AlphaFold3 predicts interactions between the calmodulin superfamily and VPS13 proteins. (A) Predicted interaction of Saccharomyces cerevisiae (*Sc*) VPS13 (light blue) with CaM (yellow) through a conserved CaM-binding domain (CaMBD, purple). (B) Predicted interaction of human VPS13D with CaM, colored as in (A). (C) Prediction of human VPS13B and CaM. The CaMBD-equivalent region between RBG_1 and RBG_2, is highlighted in purple. CaM is not predicted to interact with any part of VPS13B shown here. (D) Predicted interaction of *Sc*VPS13 with *Sc*Cdc31. *Sc*Cdc31 (orange) differs from human CaM by an additional N-terminal helix (red). (E) Predicted interaction of *Ct*VPS13 with *Ct*Cdc31. Similarly, *Ct*Cdc31 (orange) differs from human CaM by an additional N-terminal loop (red). (F) When overexpressed in Expi293F cells, *Ct*VPS13 co-purifies with CaM in FLAG-IP. The binding of calmodulin to *Ct*VPS13 is unaffected by the presence of calcium or EGTA throughout the purification. Because of the difference in sizes for *Ct*VPS13 and CaM, the same samples were run twice on different gels (4-20% Tris-glycine and 3-8% Tris-acetate) to best resolve both proteins.

bioRxiv preprint doi: https://doi.org/10.1101/2025.11.10.687702; this version posted November 11, 2025. The copyright holder for this preprint (which was not certified by peer review) is the author/funder, who has granted bioRxiv a license to display the preprint in perpetuity. It is made available under aCC-BY 4.0 International license.

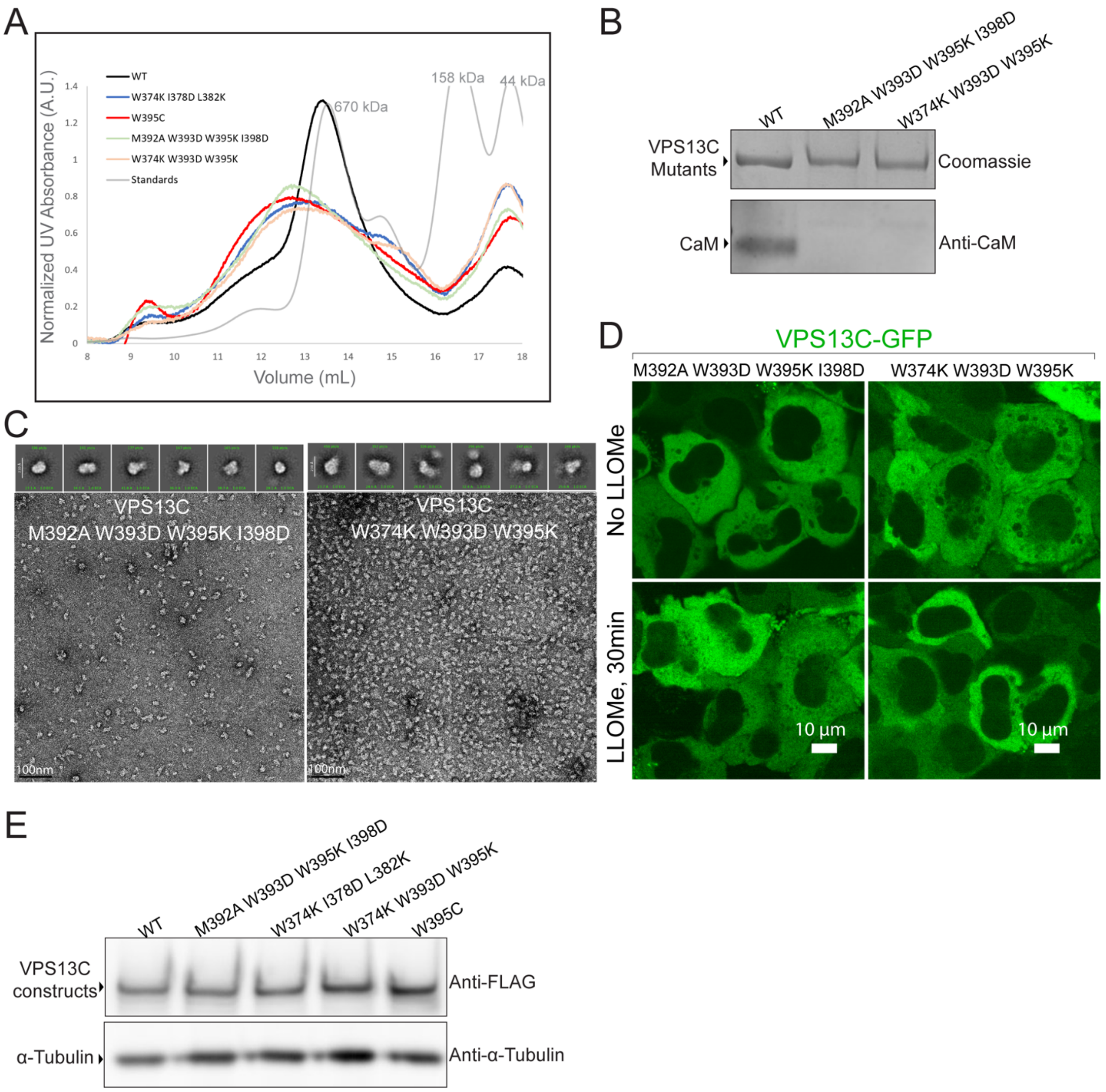

Figure S5. Characterization of calmodulin-binding defective VPS13C mutants.
(**A**) Size-exclusion chromatography of VPS13C WT and calmodulin-binding defective mutants. Mutants elute with broader and shifted peaks compared to WT, yet remain soluble and do not aggregate in the void volume. (**B**) Like W395C, additional mutants M392A/W393D/W395K/I398D and W374K/W393D/W395K do not bind CaM, (**C**) lose the rod-like shape of WT protein but remain not non-aggregated, (**D**) and fail to localize to membrane contact sites upon LLOME treatment. (**E**) All CaM-binding defective mutants are expressed at levels comparable to VPS13C WT when overexpressed in Expi293F cells.

bioRxiv preprint doi: https://doi.org/10.1101/2025.11.10.687702; this version posted November 11, 2025. The copyright holder for this preprint (which was not certified by peer review) is the author/funder, who has granted bioRxiv a license to display the preprint in perpetuity. It is made available under aCC-BY 4.0 International license.

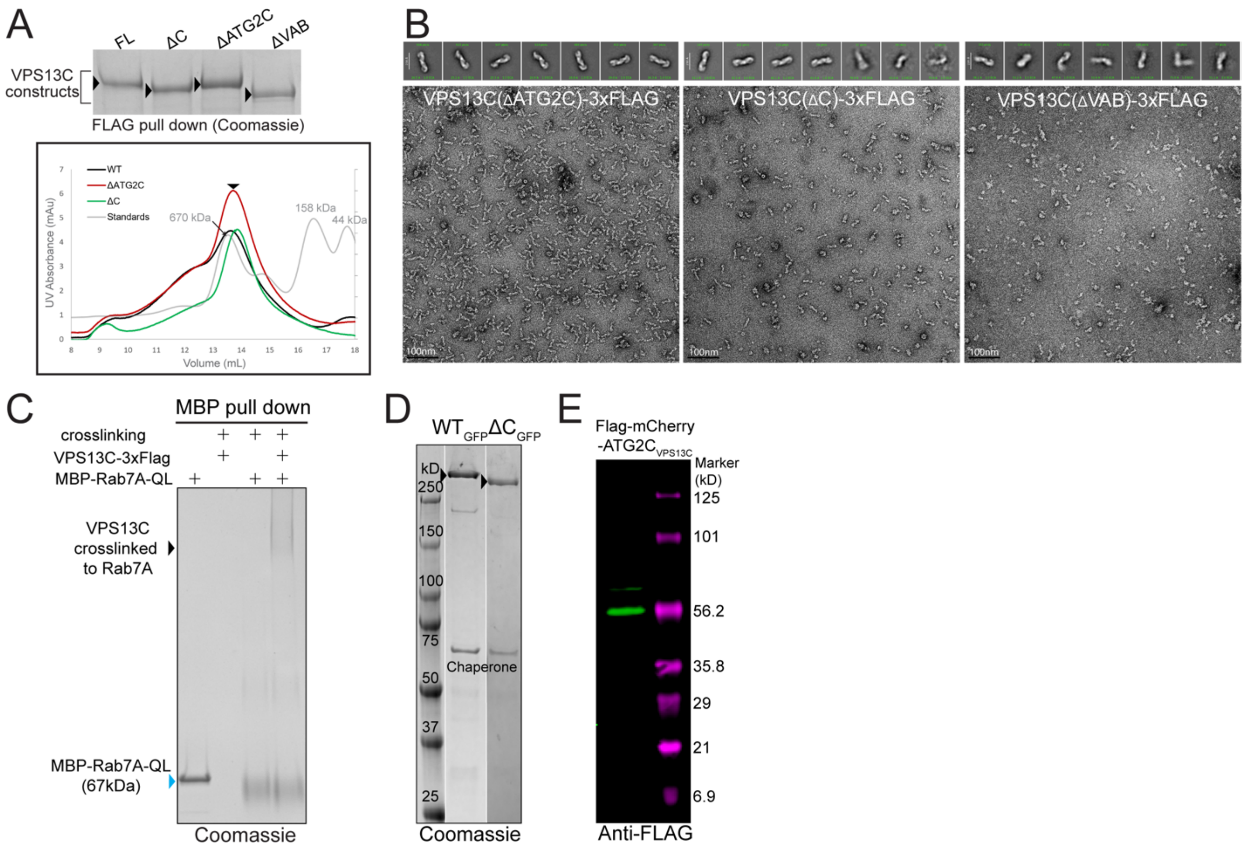

Figure S6. Quality control of VPS13C constructs by purification, EM, and crosslinking.
(**A**) VPS13C deletion constructs used for Rab7A pull down experiments and liposome flotation assays are not degraded or aggregated, and migrate at their expected molecular weights on the gel and by size-exclusion chromatography. (**B**) Representative negative-stain EM images and 2D averages show that VPS13C deletion constructs are well-folded and not aggregated. (**C**) Purified full-length FLAG-tagged VPS13C forms a complex with MBP-tagged Rab7A upon glutaldehyde crosslinking. (**D**) GFP-tagged VPS13C WT and VPS13C-ΔC used for GUV experiments are pure and intact, not degraded. (**E)** mCherry-tagged VPS13C-ATG2C used for GUV experiments is intact in cell lysate.

bioRxiv preprint doi: https://doi.org/10.1101/2025.11.10.687702; this version posted November 11, 2025. The copyright holder for this preprint (which was not certified by peer review) is the author/funder, who has granted bioRxiv a license to display the preprint in perpetuity. It is made available under aCC-BY 4.0 International license.

Supplementary Table S1. Plasmids used in this study.

No.	Name	Insert	Residue number and mutations	Addgene #
1	pCAG-VPS13C-WT-3xFLAG	VPS13C	1-3753	248315
2	pCAG-VPS13C-ΔATG2C-3xFLAG	VPS13C	1-3476-GS-3585-3753	248316
3	pCAG-VPS13C-ΔC-3xFLAG	VPS13C	1-3418	248317
4	pCAG-VPS13C-ΔVAB-3xFLAG	VPS13C	1-2411-3x(GGGGS)-3068-3753	248318
5	pCAG-VPS13C-ΔCΔWWE-3xFLAG	VPS13C	1-3116; 3185-3418	248319
6	pCAG-VPS13C-ΔATG2CΔVAB-3xFLAG	VPS13C	1-2411-3x(GGGGS)-3068-3476-GS-3585-3753	248320
7	pCAG-VPS13C-VAB-3xFLAG	VPS13C	2422-3083	248321
8	pCMV10-VPS13C-WT-GFP-3xFLAG	VPS13C	1-3753	248322
9	pCMV10-VPS13C-M392A W393D W395K I398D-GFP-3xFLAG	VPS13C	1-3753(M392A W393D W395K I398D)	248323
10	pCMV10-VPS13C-W374K I378D L382K-GFP-3xFLAG	VPS13C	1-3753(W374K I378D L382K)	248324
11	pCMV10-VPS13C-W374K W393D W395K-GFP-3xFLAG	VPS13C	1-3753(W374K W393D W395K)	248325
12	pCMV10-VPS13C-W395C-GFP-3xFLAG	VPS13C	1-3753(W395C)	248326
13	pCMV10-VPS13C-ΔC-GFP-3xFLAG	VPS13C	1-3418	248327
14	pCMV10-mCherry-ATG2C	VPS13C	3419-3609	232868
15	pCAG-Strep-Rab7-Q67L	Rab7A	1-207 (Q67L)	248328
16	pCAG-MBP-TEV-Rab7-Q67L-6His	Rab7A	1-207 (Q67L)	248329
17	pCAG- 3xFLAG-*Ct*VPS13	*Ct*VPS13	1-3225	248330
18	pETDuet-6xHis-Strep-*Ct*VPS13 (1-635)	*Ct*VPS13	1-635	248331
19	pETDuet-6xHis-Strep-*Ct*VPS13 (1-635) + 6xHis-*Hs*CaM	*Ct*VPS13, *Hs*CaM	1-635; 2-149	248332

bioRxiv preprint doi: https://doi.org/10.1101/2025.11.10.687702; this version posted November 11, 2025. The copyright holder for this preprint (which was not certified by peer review) is the author/funder, who has granted bioRxiv a license to display the preprint in perpetuity. It is made available under aCC-BY 4.0 International license.

Supplementary Table S2. Cryo-EM and model statistics.

Description	***Hs*VPS13C with co-purified human calmodulin**				***Ct*VPS13α**
	**C-terminal**	**N-terminal**	**Full-length**	**Composite**	
	PDB:9YQQ	PDB:9YQP		PDB:9YRP	PDB:9YRM
	EMDB:73344	EMDB:73343	EMDB:73345	EMDB:73373	EMDB:21113
**Data Collection and Processing**					
Facility	Yale West Campus-Cryo-EM facility				EMDB-21113
Microscope	Titan Krios				
Voltage (kV)	300				
Camera	K3				
Magnification	81,000				
Pixel Size (Å)	0.534 (super resolution)				
Total Electron Exposure (e-/A^2)	43				
Defocus Range (µm)	2-2.5				
Symmetry Imposed	C1				
Num of mics	7178				
Initial Particles	2,040,737	4,203,081	2,072,307		
Final Particles	403,001	461,163	392,473		
**Refinement**					
Initial models	AlphaFold	AlphaFold	AlphaFold and N and C-terminal		
Map pixel size	1.424	1.424	1.424	1.424	
Map Resolution (Å) (FSC	3.80	4.10	4.13		
Map sharpening B-factor (Å^2)	-111.65	-106.52	-147.66		
**Model Composition**					
Non-hydrogen atoms	13839	8956		24668	9116
Protein residues	1739	1118		3092	1128
Ligands	0	0		0	0
**Model vs. Data**					
FSC Map to Model (Å) (FSC	6.6	7.4		7.7	4.3
Correlation	0.61	0.59		0.59	0.69
**B factors (A^2)**					
Protein	27.83	33.64		53.96	97.60
**R.m.s deviation**					
Bond length (Å)	0.004	0.005		0.004	0.005
Bond angles (º)	1.006	1.064		1.049	0.727
**Validation**					
Molprobity score	2.01	2.22		2.05	2.06
Clashscore	13.68	15.69		14.94	13.88
Rotamer outliers	0.25	0		0	1.09
**Ramachandran plot**					
Outliers (%)	0.12	0		0	0
Allowed (%)	5.18	8.91		5.33	5.6
Favored (%)	94.7	91.09		94.67	94.4
**Rama-Z (whole)**	-1.71	-0.53		-1.34	-0.67

bioRxiv preprint doi: https://doi.org/10.1101/2025.11.10.687702; this version posted November 11, 2025. The copyright holder for this preprint (which was not certified by peer review) is the author/funder, who has granted bioRxiv a license to display the preprint in perpetuity. It is made available under aCC-BY 4.0 International license.

Supplementary Table S3: Top hits that co-purified with VPS13C.

#	Identified Proteins (361)	Accession Number	Alternate ID	Molecular Weight
1	Cluster of VPS13C-linker-GFP-linker-3xFlag	VPS13C-linker-GFP-linker-3xFlag [3]		454 kDa
2	Heat shock 70 kDa protein 1A OS=Homo sapiens OX=9606 GN=HSPA1A PE=1 SV=1	P0DMV8	HSPA1A	70 kDa
3	Cluster of Tubulin alpha-1A chain OS=Homo sapiens OX=9606 GN=TUBA1A PE=1 SV=1 (Q71U36)	Q71U36 [3]	TUBA1A	50 kDa
4	Cluster of Tubulin beta chain OS=Homo sapiens OX=9606 GN=TUBB PE=1 SV=2 (P07437)	P07437 [6]	TUBB	50 kDa
5	Cluster of Calmodulin-1 OS=Homo sapiens OX=9606 GN=CALM1 PE=1 SV=1 (P0DP23)	P0DP23 [2]	CALM1	17 kDa
6	Microtubule-associated protein 1B OS=Homo sapiens OX=9606 GN=MAP1B PE=1 SV=2	P46821	MAP1B	271 kDa
7	Cluster of Heat shock cognate 71 kDa protein OS=Homo sapiens OX=9606 GN=HSPA8 PE=1 SV=1 (P11142)	P11142 [3]	HSPA8	71 kDa
8	Serine/threonine-protein kinase 38 OS=Homo sapiens OX=9606 GN=STK38 PE=1 SV=1	Q15208	STK38	54 kDa
9	Cluster of Ras-related protein Rab-1A OS=Homo sapiens OX=9606 GN=RAB1A PE=1 SV=3 (P62820)	P62820 [10]	RAB1A	23 kDa
10	Serine/threonine-protein kinase 38-like OS=Homo sapiens OX=9606 GN=STK38L PE=1 SV=3	Q9Y2H1	STK38L	54 kDa
11	OTU domain-containing protein 4 OS=Homo sapiens OX=9606 GN=OTUD4 PE=1 SV=4	Q01804	OTUD4	124 kDa
12	Perilipin-3 OS=Homo sapiens OX=9606 GN=PLIN3 PE=1 SV=3	O60664	PLIN3	47 kDa
13	Cluster of Keratin, type I cytoskeletal 10 OS=Homo sapiens OX=9606 GN=KRT10 PE=1 SV=6 (P13645)	P13645 [3]	KRT10	59 kDa
14	Keratin, type II cytoskeletal 1 OS=Homo sapiens OX=9606 GN=KRT1 PE=1 SV=6	P04264	KRT1	66 kDa
15	E3 ubiquitin-protein ligase TRIM21 OS=Homo sapiens OX=9606 GN=TRIM21 PE=1 SV=1	P19474	TRIM21	54 kDa
16	Cluster of Filamin-A OS=Homo sapiens OX=9606 GN=FLNA PE=1 SV=4 (P21333)	P21333 [2]	FLNA	281 kDa
17	Cluster of Ras-related protein Rab-5C OS=Homo sapiens OX=9606 GN=RAB5C PE=1 SV=2 (P51148)	P51148 [3]	RAB5C	23 kDa
18	Heterogeneous nuclear ribonucleoprotein M OS=Homo sapiens OX=9606 GN=HNRNPM PE=1 SV=3	P52272	HNRNPM	78 kDa
19	Tetratricopeptide repeat protein 1 OS=Homo sapiens OX=9606 GN=TTC1 PE=1 SV=1	Q99614	TTC1	34 kDa
20	Keratin, type II cytoskeletal 2 epidermal OS=Homo sapiens OX=9606 GN=KRT2 PE=1 SV=2	P35908	KRT2	65 kDa
21	Actin, cytoplasmic 1 OS=Homo sapiens OX=9606 GN=ACTB PE=1 SV=1	P60709 (+1)	ACTB	42 kDa
22	Ras-related protein Rab-7a OS=Homo sapiens OX=9606 GN=RAB7A PE=1 SV=1	P51149	RAB7A	23 kDa
23	Protein arginine N-methyltransferase 5 OS=Homo sapiens OX=9606 GN=PRMT5 PE=1 SV=4	O14744	PRMT5	73 kDa
24	Cluster of Keratin, type II cytoskeletal 6B OS=Homo sapiens OX=9606 GN=KRT6B PE=1 SV=5 (P04259)	P04259 [2]	KRT6B	60 kDa
25	ADP-ribosylation factor GTPase-activating protein 1 OS=Homo sapiens OX=9606 GN=ARFGAP1 PE=1 SV=2	Q8N6T3	ARFGAP1	45 kDa
26	Heat shock protein HSP 90-alpha OS=Homo sapiens OX=9606 GN=HSP90AA1 PE=1 SV=5	P07900	HSP90AA1	85 kDa
27	Cluster of Heat shock protein HSP 90-beta OS=Homo sapiens OX=9606 GN=HSP90AB1 PE=1 SV=4 (P08238)	P08238 [2]	HSP90AB1	83 kDa
28	Melanoma-associated antigen D2 OS=Homo sapiens OX=9606 GN=MAGED2 PE=1 SV=2	Q9UNF1	MAGED2	65 kDa
29	Exportin-2 OS=Homo sapiens OX=9606 GN=CSE1L PE=1 SV=3	P55060	CSE1L	110 kDa
30	Polyubiquitin-B OS=Homo sapiens OX=9606 GN=UBB PE=1 SV=1	P0CG47 (+3)	UBB	26 kDa
31	Cluster of Serpin B3 OS=Homo sapiens OX=9606 GN=SERPINB3 PE=1 SV=2 (P29508)	P29508 [2]	SERPINB3	45 kDa
32	Cluster of CLIP-associating protein 2 OS=Homo sapiens OX=9606 GN=CLASP2 PE=1 SV=3 (O75122)	O75122 [2]	CLASP2	141 kDa

bioRxiv preprint doi: https://doi.org/10.1101/2025.11.10.687702; this version posted November 11, 2025. The copyright holder for this preprint (which was not certified by peer review) is the author/funder, who has granted bioRxiv a license to display the preprint in perpetuity. It is made available under aCC-BY 4.0 International license.

33	Protein S100-A9 OS=Homo sapiens OX=9606 GN=S100A9 PE=1 SV=1	P06702	S100A9	13 kDa
34	Protein S100-A7 OS=Homo sapiens OX=9606 GN=S100A7 PE=1 SV=4	P31151	S100A7	11 kDa
35	Poly(rC)-binding protein 1 OS=Homo sapiens OX=9606 GN=PCBP1 PE=1 SV=2	Q15365	PCBP1	37 kDa
36	Ras-related protein Rab-11B OS=Homo sapiens OX=9606 GN=RAB11B PE=1 SV=4	Q15907	RAB11B	24 kDa
37	Tumor protein D54 OS=Homo sapiens OX=9606 GN=TPD52L2 PE=1 SV=2	O43399	TPD52L2	22 kDa
38	BAG family molecular chaperone regulator 2 OS=Homo sapiens OX=9606 GN=BAG2 PE=1 SV=1	O95816	BAG2	24 kDa
39	Keratin, type I cytoskeletal 9 OS=Homo sapiens OX=9606 GN=KRT9 PE=1 SV=3	P35527	KRT9	62 kDa
40	Kinesin-like protein KIF11 OS=Homo sapiens OX=9606 GN=KIF11 PE=1 SV=2	P52732	KIF11	119 kDa
41	Small ribosomal subunit protein eS4, X isoform OS=Homo sapiens OX=9606 GN=RPS4X PE=1 SV=2	P62701	RPS4X	30 kDa
42	Spartin OS=Homo sapiens OX=9606 GN=SPART PE=1 SV=1	Q8N0X7	SPART	73 kDa
43	c-Myc-binding protein OS=Homo sapiens OX=9606 GN=MYCBP PE=1 SV=3	Q99417	MYCBP	12 kDa
44	RuvB-like 2 OS=Homo sapiens OX=9606 GN=RUVBL2 PE=1 SV=3	Q9Y230	RUVBL2	51 kDa
45	Exportin-1 OS=Homo sapiens OX=9606 GN=XPO1 PE=1 SV=1	O14980	XPO1	123 kDa
46	Protein S100-A8 OS=Homo sapiens OX=9606 GN=S100A8 PE=1 SV=1	P05109	S100A8	11 kDa
47	Cluster of 14-3-3 protein gamma OS=Homo sapiens OX=9606 GN=YWHAG PE=1 SV=2 (P61981)	P61981 [4]	YWHAG	28 kDa
48	Large ribosomal subunit protein eL24 OS=Homo sapiens OX=9606 GN=RPL24 PE=1 SV=1	P83731	RPL24	18 kDa
49	Nucleolar protein 56 OS=Homo sapiens OX=9606 GN=NOP56 PE=1 SV=4	O00567	NOP56	66 kDa
50	Large ribosomal subunit protein eL13 OS=Homo sapiens OX=9606 GN=RPL13 PE=1 SV=4	P26373	RPL13	24 kDa

bioRxiv preprint doi: https://doi.org/10.1101/2025.11.10.687702; this version posted November 11, 2025. The copyright holder for this preprint (which was not certified by peer review) is the author/funder, who has granted bioRxiv a license to display the preprint in perpetuity. It is made available under aCC-BY 4.0 International license.

Supplementary Table S4: Key lab materials used and generated in this study.

Dataset	Raw Cryo-EM data	EMPAIR	EMPIAR-13070	NEW	
Dataset	All source data generated in this study	Zenodo	https://doi.org/10.5281/zenodo.17417584	NEW	
Dataset	All tables used in this study	Zenodo	https://doi.org/10.5281/zenodo.17459272	NEW	
Dataset	CryoEM map of the N-terminal VPS13C	EMDB	EMD-73343	NEW	
Dataset	Model built for N-terminal VPS13C	PDB	PDB-9YQP	NEW	
Dataset	Cryo-EM map of the C-terminal VPS13C	EMDB	EMD-73344	NEW	
Dataset	Model built for C-terminal VPS13C	PDB	PDB-9YQQ	NEW	
Dataset	Concensus CryoEM map of full-length VPS13C	EMDB	EMD-73345	NEW	
Dataset	Composite map of full-length VPS13C	EMDB	EMD-73373	NEW	
Dataset	Model built for composite full-length VPS13C	PDB	PDB-9YRP	NEW	
Dataset	Model built for EMDB-21113, VPS13(1-1390) from C.thermphilium	PDB	PDB-9YRM	NEW	
Software	CryoSPARC Version 4.6.2	Structura Biotechnology	https://cryosparc.com/;RRID:SCR_016501	REUSE	
Software	ImageJ Version 1.52e	National Institute of Health (NIH)	https://imagej.net/ij/;RRID:SCR_003070	REUSE	
Software	WinCoot 1.1.14	MRC-LMB	https://www2.mrc-lmb.cam.ac.uk/personal/pemsley/coot/; RRID:SCR_014222	REUSE	
Software	Phenix 1.21.2-5419	Phenix	https://phenix-online.org/; RRID:SCR_014224	REUSE	
Software	ChimeraX 1.9	UCSF	https://www.rbvi.ucsf.edu/chimerax/; RRID:SCR_015872	REUSE	
Software	SerialEM	SerialEM	https://bio3d.colorado.edu/SerialEM/; RRID:SCR_017293	REUSE	
Software	AlphaFold 3	AlphaFold Server	https://alphafoldserver.com/about; RRID:SCR_025885	REUSE	
Software	DeepMainMast	Kiharalab EMSuite Server	https://em.kiharalab.org/algorithm/DeepMainMast	REUSE	
Software	Namdinator	Namdinator Server	https://namdinator.au.dk/	REUSE	
Software	spIsoNet	spIsoNet	https://github.com/IsoNet-cryoET/spIsoNet	REUSE	
Software	GraphPad Prism 6	GraphPad Prism	http://www.graphpad.com/; RRID:SCR_002798	REUSE	
Software	Pymol 2.6.0a0	Schrödinger, LLC	https://www.pymol.org/; RRID:SCR_000305	REUSE	
Protocol	Endogenous calmodulin co-purified with VPS13s detected by western blotting	protocol.io	https://doi.org/10.17504/protocols.io.kqdg31w17l25/v1	NEW	
Protocol	Cell culture, transfection, immunocytochemistry, and imaging	protocol.io	https://doi.org/10.17504/protocols.io.eq2lyp55mlx9/v1	REUSE	
Protocol	Expression, purification, and characeraization of VPS13C	protocol.io	https://doi.org/10.17504/protocols.io.rm7vz92d4gx1/v1	NEW	
Protocol	Cryo-EM structural determination of VPS13C	protocol.io	https://doi.org/10.17504/protocols.io.36wgqpqpovk5/v1	NEW	
Protocol	Expression, purification, and characterization of CtVPS13(1-635) with calmodulin	protocol.io	https://doi.org/10.17504/protocols.io.ewov11m17vr2/v1	NEW	
Protocol	Complex formation between VPS13C and Rab7	protocol.io	https://doi.org/10.17504/protocols.io.eq2ly4q4qlx9/v1	NEW	
Protocol	Co-floatation assay of VPS13C with SUVs	protocol.io	https://doi.org/10.17504/protocols.io.q26g7n9nqlwz/v1	NEW	
Protocol	Characterization of VPS13C's binding to GUVs	protocol.io	https://doi.org/10.17504/protocols.io.81wgbwko1gpk/v1	NEW	
Antibody	Rabbit anti-calmodulin	Med Chem Express	Cat. #HY-P82082; RRID:AB_3104023	REUSE	1:1000 dilution
Antibody	Goat anti-rabbit HRP conjugate	Sigma Aldrich	Cat. #AP307P; RRID:AB_92641	REUSE	1:1000 dilution
Antibody	Mouse anti-FLAG	Sigma Aldrich	Cat. #F1804; RRID:AB_262044	REUSE	1:1000 dilution
Antibody	Goat anti-mouse HRP conjugate	Thermo Scientific	Cat. #62-6520; RRID:AB_88369	REUSE	1:1000 dilution
Antibody	Rabbit anti α-Tubulin	Cell Signaling Technology	Cat. #2125; RRID:AB_2619646	REUSE	1:1000 dilution
Antibody	Goat anto-mouse antibody conjugated to IRDye 800CW	LI-COR	Cat. #926-32350; RRID:AB_2782997	REUSE	1:10000 dilution
Cell line	HeLaM cells	N/A	Cat. #RCB5388; RRID: CVCL_R965	REUSE	
Cell line	Expi293F cells	Thermo Scientific	Cat. #A14527; RRID:CVCL_D615	REUSE	
Bacterial strain	BL21(DE3) pLysS	Agilent	Cat. #200132	REUSE	
Peptide	3xFLAG peptide	Apex Bio	Cat. #A6002	REUSE	
Recombinant protein	His-ATG7	Thomas Melia Lab, Yale University	N/A	REUSE	
Recombinant protein	Recombinant calmodulin	G-Biosciences	Cat. #786-1244	REUSE	
Plasmid	pCAG-VPS13C-WT-3xFLAG	Reinisch Lab, Yale University	RRID:Addgene_248315	NEW	
Plasmid	pCAG-VPS13C-delATG2C-3xFLAG	Reinisch Lab, Yale University	RRID:Addgene_248316	NEW	

bioRxiv preprint doi: https://doi.org/10.1101/2025.11.10.687702; this version posted November 11, 2025. The copyright holder for this preprint (which was not certified by peer review) is the author/funder, who has granted bioRxiv a license to display the preprint in perpetuity. It is made available under aCC-BY 4.0 International license.

Plasmid	pCAG-VPS13C-delC-3xFLAG	Reinisch Lab, Yale University	RRID:Addgene_248317	NEW	
Plasmid	pCAG-VPS13C-delVAB-3xFLAG	Reinisch Lab, Yale University	RRID:Addgene_248318	NEW	
Plasmid	pCAG-VPS13C-delCdelWWE-3xFLAG	Reinisch Lab, Yale University	RRID:Addgene_248319	NEW	
Plasmid	pCAG-VPS13C-delVABdelATG2C-3xFLAG	Reinisch Lab, Yale University	RRID:Addgene_248320	NEW	
Plasmid	pCAG-VPS13C-VAB-3xFLAG	Reinisch Lab, Yale University	RRID:Addgene_248321	NEW	
Plasmid	pCMV10-VPS13C-WT-GFP-3xFLAG	De Camilli Lab, Yale University	RRID:Addgene_248322	NEW	
Plasmid	pCMV10-VPS13C-M392A W393D W395K I398D-GFP-3xFLAG	Reinisch Lab, Yale University	RRID:Addgene_248323	NEW	
Plasmid	pCMV10-VPS13C-W374K I378D L382K-GFP-3xFLAG	Reinisch Lab, Yale University	RRID:Addgene_248324	NEW	
Plasmid	pCMV10-VPS13C-W374K W393D W395K-GFP-3xFLAG	Reinisch Lab, Yale University	RRID:Addgene_248325	NEW	
Plasmid	pCMV10-VPS13C-W395C-GFP-3xFLAG	Reinisch Lab, Yale University	RRID:Addgene_248326	NEW	
Plasmid	pCMV10-VPS13C-delC-GFP-3xFLAG	De Camilli Lab, Yale University	RRID:Addgene_248327	NEW	
Plasmid	pCAG-Strep-Rab7-Q67L	Reinisch Lab, Yale University	RRID:Addgene_248328	NEW	
Plasmid	pCAG-MBP-TEV-Rab7-Q67L-6His	Reinisch Lab, Yale University	RRID:Addgene_248329	NEW	
Plasmid	pCAG- 3xFLAG-CtVPS13	Reinisch Lab, Yale University	RRID:Addgene_248330	NEW	
Plasmid	pETDuet-6xHis-Strep-CtVPS13 (1-635)	Reinisch Lab, Yale University	RRID:Addgene_248331	NEW	
Plasmid	pETDuet-6xHis-Strep-CtVPS13 (1-635) + 6xHis-HsCaM	Reinisch Lab, Yale University	RRID:Addgene_248332	NEW	
Plasmid	pCMV10-mCherry-ATG2C (VPS13C)	De Camilli Lab, Yale University	RRID:Addgene_232868	REUSE	
Chemical	Protease inhibitor cocktail	Roche	Cat. #11873580001	REUSE	
Chemical	GTP (guanosine triphosphate)	Sigma	Cat. #G8877; CAS: 36051-31-7	REUSE	
Chemical	Imidazole	Sigma	Cat. #56750; CAS: 288-32-4	REUSE	
Chemical	D-Biotin	G-Biosciences	Cat. #BG-oo; CAS: 58-85-5	REUSE	
Chemical	Maltose	Sigma	Cat. #M5895; CAS: 6363-53-7	REUSE	
Chemical	Glutaraldehyde (GA)	Sigma	Cat. #G6257; CAS: 111-30-8	REUSE	
Chemical	OptiPrep (iodixanol solution)	Sigma	Cat. #D1556; CAS: 92339-11-2	REUSE	
Chemical	Opti-MEM Reduced Serum Medium	Gibco	Cat. #31985070	REUSE	
Chemical	ExpiFectamine 293 Reagent	Gibco	Cat. #A14525	REUSE	
Chemical	FLAG M2 resin	Millipore-Sigma	Cat. #A2220	REUSE	
Chemical	Talon Metal Affinity Resin	Takara	Cat. #635502	REUSE	
Chemical	Amylose Resin	NEB	Cat. #E8021	REUSE	
Chemical	Strep-Tactin XT 4Flow High-Capacity Resin	IBA	Cat. #2-5030-002	REUSE	
Chemical	ECL substrate	Thermo Scientific	Cat. #32106	REUSE	
Chemical	Rhodamine B-PE (Rhod-PE)	Avanti Polar Lipids	Cat. #810150; CAS:384833-00-5	REUSE	
Chemical	POPC (1-palmitoyl-2-oleoyl-sn-glycero-3-phosphocholine)	Avanti Polar Lipids	Cat. #850457; CAS: 26853-31-6	REUSE	
Chemical	POPE (1-palmitoyl-2-oleoyl-sn-glycero-3-phosphoethanolamine)	Avanti Polar Lipids	Cat. #850757; CAS: 26662-94-2	REUSE	
Chemical	DGS-NTA(Ni)	Avanti Polar Lipids	Cat. #790404; CAS: 231615-77-3	REUSE	
Chemical	DOPS (1,2-dioleoyl-sn-glycero-3-phospho-L-serine)	Avanti Polar Lipids	Cat. #840035; CAS: 90693-88-2	REUSE	
Chemical	Cy5-DOPE (Cyanine-5 DOPE)	Avanti Polar Lipids	Cat. #810335 ; CAS: 2260669-61-0	REUSE	
Chemical	DOG (1,2-dioleoyl-sn-glycerol)	Avanti Polar Lipids	Cat. #800811 ; CAS: 24529-88-2	REUSE	
Chemical	Ammonium persulfate (APS)	Sigma	Cat. #248614; CAS: 7727-54-0	REUSE	
Chemical	TEMED (N,N,N′,N′-Tetramethylethylenediamine)	Bio-Rad	Cat. #1610801; CAS: 110-18-9	REUSE	
Chemical	Acrylamide (AA; 40% w/v)	Sigma	Cat. #A4058; CAS: 79-06-1	REUSE	
Chemical	N,N′-Methylenebisacrylamide (BAA; 2% w/v)	Sigma	Cat. #M1533; CAS: 110-26-9	REUSE	

bioRxiv preprint doi: https://doi.org/10.1101/2025.11.10.687702; this version posted November 11, 2025. The copyright holder for this preprint (which was not certified by peer review) is the author/funder, who has granted bioRxiv a license to display the preprint in perpetuity. It is made available under aCC-BY 4.0 International license.

Chemical	(3-Aminopropyl)trimethoxysilane (APTES)	Sigma	Cat. #A3648; CAS: 919-30-2	REUSE	
Chemical	FuGene HD Transfection Reagent	Promega	Cat. #E2311	REUSE	
Chemical	DMEM (Dulbecco's Modified Eagle Medium)	Gibco	Cat. #11965092	REUSE	
Chemical	Fetal Bovine Serum (FBS)	Sigma	Cat. #F4135	REUSE	

References in legends:

Abramson, J., Adler, J., Dunger, J., Evans, R., Green, T., Pritzel, A., Ronneberger, O., Willmore, L., Ballard, A.J., Bambrick, J., *et al.* (2024). Accurate structure prediction of biomolecular interactions with AlphaFold 3. Nature *630*, 493-500.

Afonine, P.V., Poon, B.K., Read, R.J., Sobolev, O.V., Terwilliger, T.C., Urzhumtsev, A., and Adams, P.D. (2018). Real-space refinement in PHENIX for cryo-EM and crystallography. Acta Crystallogr D Struct Biol *74*, 531-544.

Andrews, C., Xu, Y., Kirberger, M., and Yang, J.J. (2020). Structural Aspects and Prediction of Calmodulin-Binding Proteins. Int J Mol Sci *22*.

Ashkenazy, H., Abadi, S., Martz, E., Chay, O., Mayrose, I., Pupko, T., and Ben-Tal, N. (2016). ConSurf 2016: an improved methodology to estimate and visualize evolutionary conservation in macromolecules. Nucleic Acids Res *44*, W344-350.

Casanal, A., Lohkamp, B., and Emsley, P. (2020). Current developments in Coot for macromolecular model building of Electron Cryo-microscopy and Crystallographic Data. Protein Sci *29*, 1069-1078.

Chen, S., McMullan, G., Faruqi, A.R., Murshudov, G.N., Short, J.M., Scheres, S.H., and Henderson, R. (2013). High-resolution noise substitution to measure overfitting and validate resolution in 3D structure determination by single particle electron cryomicroscopy. Ultramicroscopy *135*, 24-35.

Chen, W., Motsinger, M.M., Li, J., Bohannon, K.P., and Hanson, P.I. (2024). Ca(2+)-sensor ALG-2 engages ESCRTs to enhance lysosomal membrane resilience to osmotic stress. Proc Natl Acad Sci U S A *121*, e2318412121.

De, M., Oleskie, A.N., Ayyash, M., Dutta, S., Mancour, L., Abazeed, M.E., Brace, E.J., Skiniotis, G., and Fuller, R.S. (2017). The Vps13p-Cdc31p complex is directly required for TGN late endosome transport and TGN homotypic fusion. J Cell Biol *216*, 425-439.

Dziurdzik, S.K., and Conibear, E. (2021). The Vps13 Family of Lipid Transporters and Its Role at Membrane Contact Sites. Int J Mol Sci *22*.

Gahlot, P., Kravic, B., Rota, G., van den Boom, J., Levantovsky, S., Schulze, N., Maspero, E., Polo, S., Behrends, C., and Meyer, H. (2024). Lysosomal damage sensing and lysophagy initiation by SPG20-ITCH. Mol Cell *84*, 1556-1569 e1510.

Gillingham, A.K., Bertram, J., Begum, F., and Munro, S. (2019). In vivo identification of GTPase interactors by mitochondrial relocalization and proximity biotinylation. Elife *8*.

Gimenez-Andres, M., Copic, A., and Antonny, B. (2018). The Many Faces of Amphipathic Helices. Biomolecules *8*.

Goddard, T.D., Huang, C.C., Meng, E.C., Pettersen, E.F., Couch, G.S., Morris, J.H., and Ferrin, T.E. (2018). UCSF ChimeraX: Meeting modern challenges in visualization and analysis. Protein Sci *27*, 14-25.

bioRxiv preprint doi: https://doi.org/10.1101/2025.11.10.687702; this version posted November 11, 2025. The copyright holder for this preprint (which was not certified by peer review) is the author/funder, who has granted bioRxiv a license to display the preprint in perpetuity. It is made available under aCC-BY 4.0 International license.

Guillen-Samander, A., Leonzino, M., Hanna, M.G., Tang, N., Shen, H., and De Camilli, P. (2021). VPS13D bridges the ER to mitochondria and peroxisomes via Miro. J Cell Biol *220*.

Hanna, M., Guillen-Samander, A., and De Camilli, P. (2023). RBG Motif Bridge-Like Lipid Transport Proteins: Structure, Functions, and Open Questions. Annu Rev Cell Dev Biol *39*, 409-434.

Jumper, J., Evans, R., Pritzel, A., Green, T., Figurnov, M., Ronneberger, O., Tunyasuvunakool, K., Bates, R., Zidek, A., Potapenko, A., *et al.* (2021). Highly accurate protein structure prediction with AlphaFold. Nature *596*, 583-589.

Jurado, L.A., Chockalingam, P.S., and Jarrett, H.W. (1999). Apocalmodulin. Physiol Rev *79*, 661-682.

Kang, Y., Lehmann, K.S., Long, H., Jefferson, A., Purice, M., Freeman, M., and Clark, S. (2025). Structural basis of lipid transfer by a bridge-like lipid-transfer protein. Nature *642*, 242-249.

Kidmose, R.T., Juhl, J., Nissen, P., Boesen, T., Karlsen, J.L., and Pedersen, B.P. (2019). Namdinator - automatic molecular dynamics flexible fitting of structural models into cryo-EM and crystallography experimental maps. IUCrJ *6*, 526-531.

Kilmartin, J.V. (2003). Sfi1p has conserved centrin-binding sites and an essential function in budding yeast spindle pole body duplication. J Cell Biol *162*, 1211-1221.

Kors, S., Costello, J.L., and Schrader, M. (2022). VAP Proteins - From Organelle Tethers to Pathogenic Host Interactors and Their Role in Neuronal Disease. Front Cell Dev Biol *10*, 895856.

Kumar, N., Leonzino, M., Hancock-Cerutti, W., Horenkamp, F.A., Li, P., Lees, J.A., Wheeler, H., Reinisch, K.M., and De Camilli, P. (2018). VPS13A and VPS13C are lipid transport proteins differentially localized at ER contact sites. J Cell Biol *217*, 3625-3639.

Lesage, S., Drouet, V., Majounie, E., Deramecourt, V., Jacoupy, M., Nicolas, A., Cormier-Dequaire, F., Hassoun, S.M., Pujol, C., Ciura, S., *et al.* (2016). Loss of VPS13C Function in Autosomal-Recessive Parkinsonism Causes Mitochondrial Dysfunction and Increases PINK1/Parkin-Dependent Mitophagy. Am J Hum Genet *98*, 500-513.

Levine, T.P. (2022). Sequence Analysis and Structural Predictions of Lipid Transfer Bridges in the Repeating Beta Groove (RBG) Superfamily Reveal Past and Present Domain Variations Affecting Form, Function and Interactions of VPS13, ATG2, SHIP164, Hobbit and Tweek. Contact (Thousand Oaks) *5*, 251525642211343.

Li, P., Lees, J.A., Lusk, C.P., and Reinisch, K.M. (2020). Cryo-EM reconstruction of a VPS13 fragment reveals a long groove to channel lipids between membranes. J Cell Biol *219*.

Liu, Y.T., Fan, H., Hu, J.J., and Zhou, Z.H. (2025). Overcoming the preferred-orientation problem in cryo-EM with self-supervised deep learning. Nat Methods *22*, 113-123.

Lloyd-Evans, E., and Waller-Evans, H. (2020). Lysosomal Ca(2+) Homeostasis and Signaling in Health and Disease. Cold Spring Harb Perspect Biol *12*.

Mastronarde, D.N. (2005). Automated electron microscope tomography using robust prediction of specimen movements. J Struct Biol *152*, 36-51.

Punjani, A., Rubinstein, J.L., Fleet, D.J., and Brubaker, M.A. (2017). cryoSPARC: algorithms for rapid unsupervised cryo-EM structure determination. Nat Methods *14*, 290-296.

Rampoldi, L., Dobson-Stone, C., Rubio, J.P., Danek, A., Chalmers, R.M., Wood, N.W., Verellen, C., Ferrer, X., Malandrini, A., Fabrizi, G.M., *et al.* (2001). A conserved sorting-associated protein is mutant in chorea-acanthocytosis. Nat Genet *28*, 119-120.

bioRxiv preprint doi: https://doi.org/10.1101/2025.11.10.687702; this version posted November 11, 2025. The copyright holder for this preprint (which was not certified by peer review) is the author/funder, who has granted bioRxiv a license to display the preprint in perpetuity. It is made available under aCC-BY 4.0 International license.

Reinisch, K.M., De Camilli, P., and Melia, T.J. (2025). Lipid Dynamics at Membrane Contact Sites. Annu Rev Biochem *94*, 479-502.

Rosenthal, P.B., and Henderson, R. (2003). Optimal determination of particle orientation, absolute hand, and contrast loss in single-particle electron cryomicroscopy. J Mol Biol *333*, 721-745.

Schneider, C.A., Rasband, W.S., and Eliceiri, K.W. (2012). NIH Image to ImageJ: 25 years of image analysis. Nat Methods *9*, 671-675.

Shen, X., Valencia, C.A., Gao, W., Cotten, S.W., Dong, B., Huang, B.C., and Liu, R. (2008). Ca(2+)/Calmodulin-binding proteins from the C. elegans proteome. Cell Calcium *43*, 444-456.

Soczewka, P., Kolakowski, D., Smaczynska-de Rooij, I., Rzepnikowska, W., Ayscough, K.R., Kaminska, J., and Zoladek, T. (2019). Yeast-model-based study identified myosin- and calcium-dependent calmodulin signalling as a potential target for drug intervention in chorea-acanthocytosis. Dis Model Mech *12*.

Song, Y., DiMaio, F., Wang, R.Y., Kim, D., Miles, C., Brunette, T., Thompson, J., and Baker, D. (2013). High-resolution comparative modeling with RosettaCM. Structure *21*, 1735-1742.

Terashi, G., Wang, X., Prasad, D., Nakamura, T., and Kihara, D. (2024). DeepMainmast: integrated protocol of protein structure modeling for cryo-EM with deep learning and structure prediction. Nat Methods *21*, 122-131.

Ueno, S., Maruki, Y., Nakamura, M., Tomemori, Y., Kamae, K., Tanabe, H., Yamashita, Y., Matsuda, S., Kaneko, S., and Sano, A. (2001). The gene encoding a newly discovered protein, chorein, is mutated in chorea-acanthocytosis. Nat Genet *28*, 121-122.

Vamparys, L., Gautier, R., Vanni, S., Bennett, W.F., Tieleman, D.P., Antonny, B., Etchebest, C., and Fuchs, P.F. (2013). Conical lipids in flat bilayers induce packing defects similar to that induced by positive curvature. Biophys J *104*, 585-593.

Wang, X., Xu, P., Bentley-DeSousa, A., Hancock-Cerutti, W., Cai, S., Johnson, B.T., Tonelli, F., Shao, L., Talaia, G., Alessi, D.R., *et al.* (2025). The bridge-like lipid transport protein VPS13C/PARK23 mediates ER-lysosome contacts following lysosome damage. Nat Cell Biol *27*, 776-789.

Wang, Y., Dahmane, S., Ti, R., Mai, X., Zhu, L., Carlson, L.A., and Stjepanovic, G. (2024). Structural basis for lipid transfer by the ATG2A-ATG9A complex. Nat Struct Mol Biol.

Wardaszka, P., Soczewka, P., Sienko, M., Zoladek, T., and Kaminska, J. (2021). Partial Inhibition of Calcineurin Activity by Rcn2 as a Potential Remedy for Vps13 Deficiency. Int J Mol Sci *22*.

Yan, C., Wu, F., Jernigan, R.L., Dobbs, D., and Honavar, V. (2008). Characterization of protein-protein interfaces. Protein J *27*, 59-70.

Yeshaw, W.M., van der Zwaag, M., Pinto, F., Lahaye, L.L., Faber, A.I., Gomez-Sanchez, R., Dolga, A.M., Poland, C., Monaco, A.P., van, I.S.C., *et al.* (2019). Human VPS13A is associated with multiple organelles and influences mitochondrial morphology and lipid droplet motility. Elife *8*.
